# Pan-cancer evaluation of clinical value of mitotic network activity index (MNAI) and its predictive value for immunotherapy

**DOI:** 10.3389/fonc.2023.1178568

**Published:** 2023-06-27

**Authors:** Xuanyu Mao, Yimeng Cai, Sarah Long, Jesus Perez-Losada, Jian-Hua Mao, Hang Chang

**Affiliations:** ^1^ Biological Systems and Engineering Division, Lawrence Berkeley National Laboratory, Berkeley, CA, United States; ^2^ Berkeley Biomedical Data Science Center, Lawrence Berkeley National Laboratory, Berkeley, CA, United States; ^3^ Department of Molecular and Cell Biology, University of California, Berkeley, CA, United States; ^4^ Instituto de Biología Molecular y Celular del Cáncer (IBMCC-CIC), Universidad de Salamanca/CSIC, Salamanca, Spain; ^5^ Instituto de Investigación Biosanitaria de Salamanca (IBSAL), Salamanca, Spain

**Keywords:** pan-cancer, breast cancer, mitotic network activity index, prognostic biomarker, genetic instability, immunotherapy, multimodal biomarker, cellular morphometric subtype

## Abstract

Increased mitotic activity is associated with the genesis and aggressiveness of many cancers. To assess the clinical value of mitotic activity as prognostic biomarker, we performed a pan-cancer study on the mitotic network activity index (MNAI) constructed based on 54-gene mitotic apparatus network. Our pan-cancer assessment on TCGA (33 tumor types, 10,061 patients) and validation on other publicly available cohorts (23 tumor types, 9,209 patients) confirmed the significant association of MNAI with overall survival, progression-free survival, and other prognostic endpoints in multiple cancer types, including lower-grade gliomas (LGG), breast invasive carcinoma (BRCA), as well as many others. We also showed significant association between MNAI and genetic instability, which provides a biological explanation of its prognostic impact at pan-cancer landscape. Our association analysis revealed that patients with high MNAI benefitted more from anti-PD-1 and Anti-CTLA-4 treatment. In addition, we demonstrated that multimodal integration of MNAI and the AI-empowered Cellular Morphometric Subtypes (CMS) significantly improved the predictive power of prognosis compared to using MNAI and CMS alone. Our results suggest that MNAI can be used as a potential prognostic biomarker for different tumor types toward different clinical endpoints, and multimodal integration of MNAI and CMS exceeds individual biomarker for precision prognosis.

## 1 Introduction

A high proliferation rate is one of the hallmarks of cancer (1). Cancer cells acquire the capability to enable chronic proliferation through genetic and epigenetic alterations (2). A few methods have been developed for clinical use to assess proliferative activity, such as the Ki-67 nuclear antigen and mitotic index. Mitotic index, the ratio of the number of cells undergoing mitosis to the total number of cells in a population, has been widely used in clinical practice for tumor grading and staging (3–5). Moreover, it has been successfully demonstrated to associate with patient survival in many cancer types (3, 6–8). In clinical practice, mitotic activity is typically evaluated as the number of mitotic figures in the high-power field or per fixed number of tumor cells through immunohistochemical (IHC) staining. With biotechnique advances, we seek a new approach to assess proliferative activity for prognostic impact.

Cell proliferation is tightly regulated by an intricate network of structural proteins, molecular motors, regulatory kinases, and phosphatases for error-free chromosome segregation. The ensemble of such upregulated genes that encode these proteins forms the mitotic apparatus network (9) and shows biological and clinical associations with cancer (6, 10–13). Furthermore, the association of elevated mitotic activity with the genesis and progression of many cancers encourages the development and evaluation of small molecule inhibitors of mitotic apparatus proteins as anticancer agents (6, 14). Despite the clinical trials on cancer treatment, it is also essential to evaluate the prognostic value of mitotic activity in different tumor types for potential clinical implications.

Furthermore, mitotic genes form robust transcriptional network in various tumor types in both human and animal models (15, 16), indicating the importance of mitotic network in tumor development. In this study, we define the mitotic network activity index (MNAI) as the sum of the expression of 54 genes pre-identified in the mitotic apparatus network following the same protocol as previously described (9). The use of MNAI can potentially overcome the inter-observer variations due to labor-intensive human counting, and provides an efficient and effective prognostic biomarker with clinical utility. Our previous study has shown that higher MNAI is significantly associated with poor overall survival in breast cancer (9). To further assess the clinical value of MNAI as a prognostic biomarker, we performed a pan-cancer study on MNAI. We demonstrated the association of MNAI with genetic instability at the pan-cancer level; and confirmed its prognostic value across tumor types, which covers the major cancers, including carcinoma, leukemia, lymphoma, melanoma, and tumors related to the central nervous system. Our results suggest that MNAI can be used as a potential prognostic biomarker for different tumor types toward different clinical endpoints.

## 2 Materials and methods

### 2.1 Definition of mitotic network activity index (MNAI)

MNAI is defined as the sum of the expression of 54 genes pre-identified in the mitotic apparatus network (9), including AURKA, AURKB, BUB1, CENPE, CHEK1, FOXM1, MELK, PBK, PLK1, TTK, TYMS, ASPM, BUB1B, CCNA2, CCNB1, CCNB2, CDC20, CDCA3, CDCA8, CENPA, CENPN, CEP55, DDX39A, DEPDC1, DLGAP5, EXO1, EXOSC9, FAM64A1, HJURP, KIF14, KIF18B, KIF20A, KIF23, KIF2C, KIF4A, LMNB2, MAD2L1, MCM10, MKI67, NCAPD2, NCAPG, NCAPG2, NCAPH, NDC80, PRC1, PTTG1, RFC3, RRM2, SMC4, STIL, TEX10, TPX2, UBE2S:


$$MNAI=\sum_{n=1}^{54}expression\;level\;of\;Gene\;\left(n\right)$$

Specifically, we followed the same protocol (9) during the construction of MNAI. The expression levels of 54 genes were assessed by either microarray or RNAseq, provided by the public datasets without any modification.

### 2.2 Datasets

MNAI was assessed on 10,061 patients in 33 tumor types from The Cancer Genome Atlas (TCGA) project (https://www.cbioportal.org/), including ACC, BLCA, BRCA, CESC, CHOL, COAD, DLBC, ESCA, GBM, HNSC, KICH, KIRC, LAML, LGG, LIHC, LUAD, LUSC, MESO, OV, PAAD, PCPG, PRAD, READ, SARC, SKCM, STAD, TGCT, THCA, THYM, UCEC, UCS and UVM (**Supplementary Table 1**); and validated on 28 published datasets on 23 tumor types (**Supplementary Table 2**), including three brain tumor cohorts from the Chinese Glioma Genome Atlas (CGGA) project (http://www.cgga.org.cn/); and 5 tumor types from the Kaplan-Meier Plotter database (https://kmplot.com/analysis/).

### 2.3 Biological evaluation of MNAI

The difference in MNAI between tumor and normal samples was evaluated in 25 tumor types using TNMplot (https://tnmplot.com/analysis/). Alteration frequencies of genetic variants, including mutation, chromosome structural variant, amplification, deep deletion, and multiple alterations, were evaluated in 32 tumor types in TCGA using cBioPortal. The aneuploidy score, mutation count, fraction genome altered, and tumor mutation burden (TMB) were also compared between altered and unaltered groups using cBioPortal.

### 2.4 Clinical evaluation of MNAI

The patients in each cohort were stratified into low-/high-MNAI groups using surv_cutpoint function (with default parameter settings: minprop=0.1) in survminer R package based on overall survival (OS) or progression-free survival (PFS). The prognostic value of MNAI in terms of low and high categories was evaluated using univariate Cox Proportional-Hazards Model (CoxPH) and Kaplan-Meier survival curves towards different prognostic outcomes, including OS, PFS, disease-free survival (DFS), event-free survival (EFS), metastasis-free survival (MFS), relapse-free survival (RFS) and biochemical failure-free survival (BFFS). KMPLOT (http://kmplot.com/analysis/) was used to assess the association of MNAI with beneficiaries from different immunotherapies in pan-cancer patients. Association of MNAI with PD1, PD-L1, CTLA-4 and immune cell infiltrations across tumor types in TCGA was assessed using Spearman’s correlation, where the abundance of different cell types were estimated via CIBERSORT in absolute mode (17).

### 2.5 Multimodal integration and evaluation of MNAI and cellular morphometric subtype

The patient cellular morphometric subtype (CMS) was defined by AI-empowered technique based on whole slide images of tissue histology, which has been detaily described in our previous study (18). CMS in LGG and BRCA patients were developed based on cellular morphometric biomarkers mined using machine learning techniques from TCGA-LGG and TCGA-BRCA cohorts. CMSs in these two cohorts have independent clinical values and underlying molecular associations (18, 19). Given these pre-established CMSs, the multimodal integration and evaluation were based on multivariate CoxPH model (20). Specifically, concordance index (C-index) was used to evaluate the performance of the CMS, MNAI, and integrated models with 1000 bootstrapping iterations and 80% sampling rate per iteration. Mann-Whitney non-parametric test was used for the comparison across models.

### 2.6 Statistical analysis

Pairwise strategy was used to deal with the missing data in TCGA and other cohorts in our study. For example, the assessment of the association between MNAI and OS was carried out with patients that have both MNAI and OS information, even when the PFS information was missing in some of the patients during OS evaluation. All statistical analyses were performed in R (version 3.6.0). The univariate and multivariate CoxPH model (survival package in R, version 3.2-7) and Kaplan-Meier analysis (survminer package in R, version 0.4.9) were used to assess the prognostic value of MNAI. Mann-Whitney nonparametric two-tailed test was used to test the statistical difference for continuous variables with a significant threshold of p<0.05.

## 3 Results

### 3.1 MNAI is significantly elevated in tumors compared to normal samples

**Figure 1:**
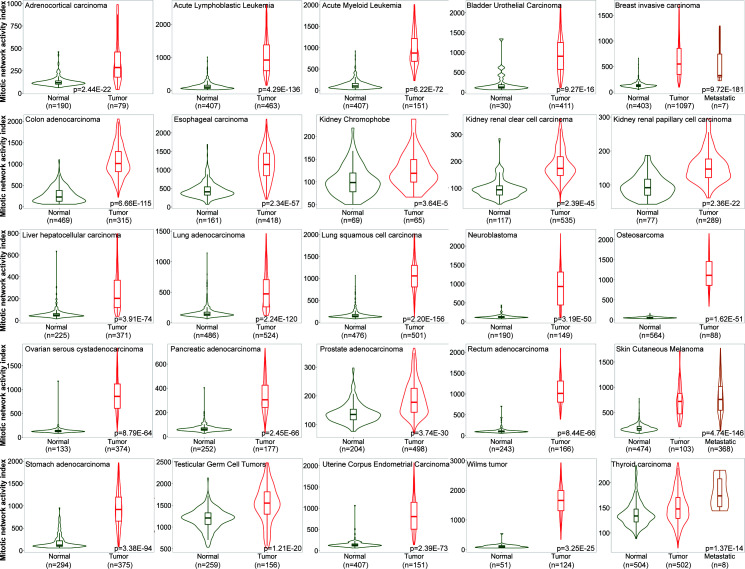
MNAI abundance is significantly elevated in cancerous tissues compared to normal tissues across different tumor types. TNMplot was used for the analysis, where Mann-Whitney non-parametric test was used to test the difference between cancerous tissues and normal tissues.

One of the major characteristics of cancerous tissue is rapid proliferation, where an increased proportion of cells undergo mitosis compared to normal ones. To confirm the power of MNAI in differentiating cancerous tissues from normal tissues, we evaluated the MNAI abundance among 25 different tumor types (**Figure 1**). Unsurprisingly, our results indicated significantly elevated MNAI in cancerous tissues among all tumor types (p< 0.0001).

### 3.2 The MNAI significantly associates with genetic instability

**Figure 2:**
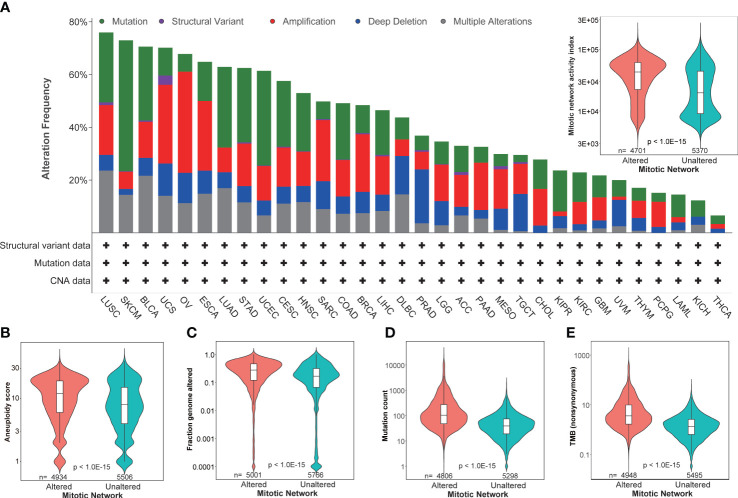
Association of MNAI with genomic instability. **(A)** Dynamic genetic alteration frequency in MNAI genes per tumor type in TCGA, including mutation, chromosome structural variant, amplification, deep deletion, and multiple alterations, where patient with any of above changes with at least one MNAI gene was classified into altered group, otherwise the patient was classified into unaltered group; **(B–E)** Association between patient groups with respect to altered and unaltered MNAI genes with aneuploidy score **(B)**, fraction genome altered **(C)**, mutation count **(D)** and tumor mutation burden **(E)**. cBioportal was used for the analysis, where Mann-Whitney non-parametric test was used to test the difference between groups.

Genomic instability is a common characteristic of most cancers, as reflected by the increased tendency of genome alteration during cell division, and is associated with increased mitotic activity (10). Our thorough evaluation based on 32 tumor types in TCGA revealed a dynamic genetic alteration frequency in MNAI genes per tumor type, including mutations, chromosome structural variants, amplifications, deep deletions, and multiple alterations. This ranged from around 80% in lung squamous cell carcinoma (LUSC) to less than 10% in thyroid carcinoma (THCA) (**Figure 2A**), Not surprisingly, MNAI in altered samples is significantly higher than in unaltered samples (**Figure 2A**, inserted panel), suggesting genetic alteration is one possible mechanism for elevated MNAI. Furthermore, significantly different aneuploidy scores (p<1.0E-15, **Figure 2B**), mutation counts (p<1.0E-15, **Figure 2C**), fraction genome altered (p<1.0E-15, **Figure 2D**), and tumor mutation burden (TMB, p<1.0E-15, **Figure 2E**) were detected at the pan-cancer level between altered and unaltered patient groups with respect to MNAI genes. Such significant difference were also observed between high and low MNAI groups within both altered and unaltered patients (**Supplementary Figure 1**), where the MNAI high/low stratification was optimized towards OS at pan-cancer level. Many existing studies have demonstrated that alteration of mitotic genes, such as AURKA, PLK1, and BUB1, induce replicative stress and mis-segregation of the chromosome, which cause aneuploidy (21, 22). Specially, one previous study showed that dysregulated transcription of MNAI genes happen prior to DNA copy number alterations (23). Together with our findings, we conclude that alteration in MNAI genes is associated with genomic instability in human cancer.

### 3.3 MNAI significantly associates with prognosis

**Figure 3:**
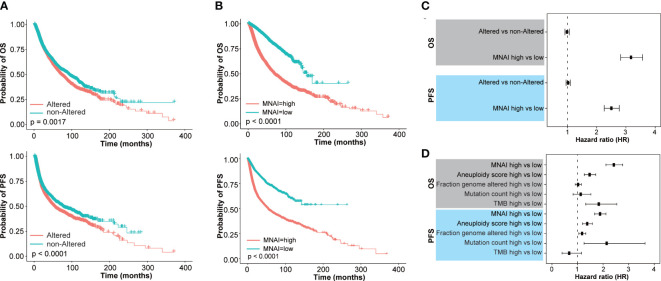
Pan-cancer prognostic value of MNAI in TCGA. **(A, B)** Kaplan-Meier plots for TCGA Pan-cancer on OS and PFS based on **(A)** genetic alteration within MNAI genes and **(B)** MNAI, where p values were calculated using log-rank test; **(C)** Hazard ratio of MNAI via univariate CoxPH analysis; **(D)** Hazard ratios of MNAI, Aneuploidy score, Fraction genome altered, Mutation count and TMB via multivariate CoxPH analysis.

Increased mitotic activity is a hallmark of cancer aggressiveness (9), and therefore associated with the prognosis of cancer patients. The evaluation and validation of MNAI on 33 different tumors confirm its significant prognostic value in the pan-cancer landscape. Specifically, in TCGA pan-cancer cohort (**Supplementary Table 1**), at pan-cancer level, both genetic alteration within MNAI genes (**Figure 3A**) and MNAI (**Figure 3B**) are significantly associated with PFS and OS. Importantly, MNAI overrode genetic alteration (**Figure 3C**) in pan-cancer prognosis. Therefore, our remaining study focused on MNAI. Consistent with previous studies, genomic instability factors significantly correlated with prognosis (**Supplementary Figure 2**), and our multivariate analysis indicates the independent and superior prognostic power of MNAI compared with genomic instability factors (**Figure 3D**).

**Figure 4:**
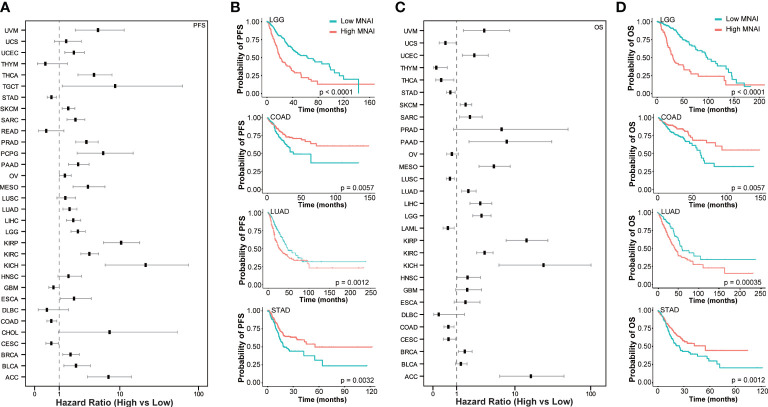
Pan-cancer prognostic value of MNAI in TCGA. **(A)** Hazard ratio of MNAI on PFS across tumor types in TCGA; **(B)** Kaplan-Meier plots of representative tumor types on PFS between high-/low-MNAI patient groups, where p values were calculated using log-rank test; **(C)** Hazard ratio of MNAI on OS across tumor types in TCGA; **(D)** Kaplan-Meier plots of representative tumor types on OS between high-/low-MNAI patient groups, where p values were calculated using log-rank test.

**Figure 5:**
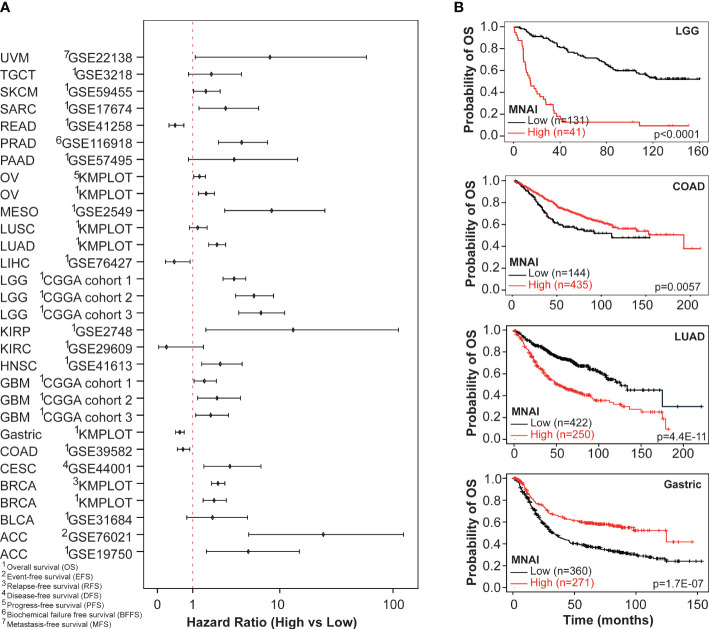
Pan-cancer prognostic value of MNAI in validation cohorts. **(A)** Hazard ratio of MNAI on different prognostic endpoints in validation cohorts; and **(B)** Kaplan-Meier plots of representative tumor types on OS between high-/low-MNAI patient groups, where p values were calculated using log-rank test.

At tumor-type-specific level, MNAI is significantly associated with PFS (**Figures 4A, B**, **Supplementary Figure 3**) and OS (**Figures 4C, D**, **Supplementary Figure 4**) of multiple tumor types, including LGG (**Figure 3B**, p<0.0001 on PFS; **Figure 3D**, p<0.0001 on OS), COAD (**Figure 4B**, p=0.0057 on PFS; **Figure 4D**, p=0.0057 on OS), LUAD (**Figure 4B**, p=0.0012 on PFS; **Figure 4D**, p=0.0035 on OS), and STAD (**Figure 4B**, p=0.0032 on PFS; **Figure 4D**, p=0.0012 on OS). Importantly, the pan-cancer prognostic value of MNAI was further confirmed on independent cohorts (**Figure 5**, **Supplementary Figure 5**, **Supplementary Table 2**), where the significant and consistent prognostic value of MNAI was observed on multiple tumor types, including LGG (**Figure 5B**, p<0.0001 on OS), COAD (**Figure 5B**, p=0.0057 on OS), LUAD (**Figure 5B**, p=4.4E-11 on OS), and Gastric (**Figure 5B**, p=1.7E-07 on OS). In addition, significant associations of MNAI with different prognostic endpoints were also observed (**Supplementary Figure 5**), including event-free survival (ACC on GSE76021, p<0.001), disease-free survival (CESC on GSE44001, p=0.0016), biochemical failure-free survival (PRAD on GSE116918, p<0.0001), metastasis-free survival (UVM on GSE22138, p=0.014), and relapse-free survival (BRCA, p<1.0E-16). Interestingly, although the high mitotic activity is typically associated with the genesis and progression of many cancers, resulting in unfavorable prognosis on TCGA datasets regarding OS (**Figure 4A**; LGG, HR=3.14, 95% CI: 2.20-4.48, p=3.0E-10; LUAD, HR=1.82, 95% CI: 1.30-2.54, p=0.00043) and PFS (**Figure 4C**; LGG, HR=2.38, 95% CI:1.79-3.16, p=2.68E-09; LUAD, HR=1.67, 95% CI: 1.22-2.29, p=0.0013). However, reversed prognosis impact of MNAI was also consistently observed on multiple tumors, including COAD and STAD with respect to both OS (**Figure 4A**; COAD, HR=0.57, 95% CI: 0.38-0.85, p=0.0064; STAD, HR=0.66, 95% CI: 0.48-0.91, p=0.012) and PFS (**Figure 4C**; COAD, HR=0.60, 95% CI: 0.41-0.86, p=0.0063; STAD, HR=0.60, 95% CI: 0.41-0.86, p=0.0035) on TCGA database as well as with OS on independent cohorts (**Figure 5A**; COAD, HR=0.65, 95% CI: 0.48-0.88, p=0.0061; STAD, HR=0.55, 95% CI: 0.43-0.69, p=1.7E-07). Such difference possibly originated from the difference in cancer biology and treatment across tumor types (24).

### 3.4 MNAI predicts the beneficial effects of immunotherapy

**Figure 6:**
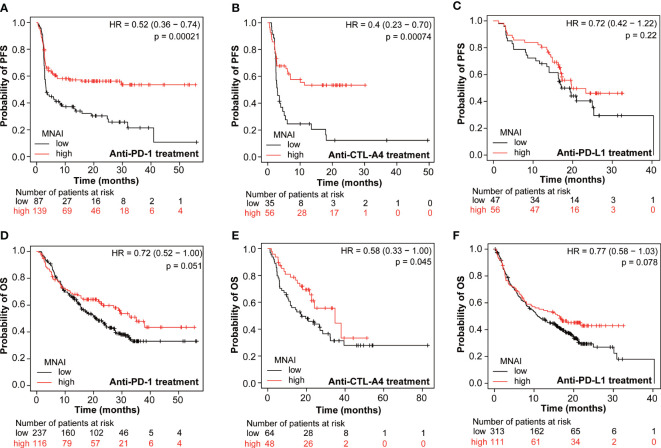
Pan-cancer predictive value of MNAI towards checkpoint inhibitor-based immunotherapy. **(A, D)** anti-PD-1 treatment of pan-cancer patients on PFS and OS, respectively. **(B, E)** anti-CTLA4 treatment of patients on PFS and OS, respectively. **(C, F)** anti-PD-L1 treatment on PFS and OS, respectively. All analyses were conducted using KMPLOT (http://kmplot.com/analysis/), where p values were calculated using log-rank test.

An increasing number of studies show that tumor mutation burden (TMB) is a predictive biomarker for immunotherapy (25, 26). Therefore, we assessed whether MNAI predicts the beneficial effects of immunotherapy. KMPlot analysis showed that cancer patients with high MNAI had significantly prolonged PFS after anti-PD-1 (p = 0.00021, **Figure 6A**) and anti-CTLA-4 (p = 0.00071, **Figure 6B**) treatment but not after anti-PD-L1 treatment (p = 0.22, **Figure 6C**). Similar observations were found for OS (**Figures 6D–F**). Utilizing the TCGA cohorts, significant association (p<0.05) was observed between MNAI and PD1, PD-L1, CTLA-4, and immune cell infiltrations found in a majority of tumor types (**Supplementary Figure 6**), which can explain its predictive powers towards certain immunotherapies. Altogether, these findings suggest that MNAI can potentially serve as a predictive biomarker for selecting patients for certain types of immunotherapies.

### 3.5 Multimodal integration of MNAI and CMS significantly improve the predictive power of prognosis

Tissue histology sections are rich in content, displaying the different cell types and overall tumor microenvironment of a given sample, and remain as the gold standard for the assessment of tissue neoplasm. To capture the cellular heterogeneity and cellular context in tissue histology, we previously defined patient subtypes as CMS using machine learning techniques from whole slide images and demonstrated its significant and independent clinical values in various tumors (18, 19). Given the translational potential of CMS and MNAI, we assessed whether the multimodal integration of MNAI and CMS further improves the predictive power of prognosis. Multivariate Cox Proportional-Hazards Model showed that the combination of CMS and MNAI significantly improved the precision prognosis in both TCGA-LGG (**Supplementary Figures 7A, B**, p<2.22E-16) and TCGA-BRCA (**Supplementary Figures 7C, D**, p<2.22E-16) cohorts towards both OS and PFS. These findings suggest that MNAI and CMS can potentially serve as a multimodal biomarker for patient risk stratification.

## 4 Discussion

In this study, we assess the prognostic and predictive value of MNAI across multiple types of human cancer. Our results showed that MNAI is consistently overexpressed in all types of cancer in comparison to their corresponding normal tissues, consistent with one of the most prominent characteristics of a cancer cell: constant proliferation. The previous study in breast cancer has shown that the upregulation of MNAI is controlled by transcription factors, including MYC (9), which may hold true over a broad range of tumors. Additionally, high MNAI values significantly correlate with increase in genome instability (aneuploidy score, fraction genome alteration, mutation count, and tumor mutation burden). It is possible that high MNAI causes mitotic errors in cell divisions, leading to the loss or gain of chromosomes and, consequently, to aneuploidy. Moreover, many MNAI genes are included in the *chromosomal* instability gene signature (13). These results elucidate mitotic genes’ potential role in genome instability and subsequent contribution to cancer development and progression.

We showed that high MNAI value in human tumors predicts poor patient outcomes, including DFS and OS in the majority of cancer types. However, we also found a favorable prognosis associated with high human tumor MNAI values in a few types of cancer. This is probably due to different first-line chemotherapies that were used in different cancer types. Patients with high MNAI only benefit from certain kinds of chemotherapies. For example, aneuploidy can make cells sensitive to certain chemotherapies but also resistant to others (27, 28). Thus, we propose that clinical implementation of the MNAI could contribute to precision cancer medicine by choosing more effective therapeutic regimens.

In recent years, immunotherapy has raised tremendous attention. Many clinical studies have shown that immune checkpoint blockade therapy can significantly improve the OS rates of patients in many types of cancer, but only in a subset of patients with cancer (29). As a result, predictive biomarkers for checkpoint inhibitor-based immunotherapy have been extensively studied, including tumor-infiltrating lymphocytes, mutational burden, immune gene signatures, and multiplex immunohistochemistry (30). In this study, we found that patients with high MNAI benefit significantly more from anti-PD-1 and Anti-CTLA-4 treatments but not from anti-PD-L1 treatment. The resistance of patients with high MNAI to anti-PD-L1 treatment is possibly due to the contribution of mitosis in cancer cells to the high expression of PD-L1 (31). These patients may potentially benefit from combined anti-mitotic and anti-PD-L1 treatments. Thus, we suggest that MNAI could serve as a novel predictive biomarker for specific checkpoint inhibitor-based therapies. Furthermore, our analysis using CancerDR database (32) revealed significant association between MNAI and many molecular targets, indicating it as a potential biomarker for predicting the response to more cancer treatment targets besides PD-1 and CTLA-4 (**Supplementary Figure 8** lower pane, spearman correlation |R|>0.3 and p value< 0.05).

In addition, CMS extracted from whole slide images of tissue histology through advanced machine learning techniques (33) has emerged as a novel imaging biomarker in various tumor types, providing independent clinical values (18, 19). In this study, we found that the multimodal integration of MNAI and CMS exceeds the individual biomarker in precision prognosis. Thus, we suggest that MNAI and CMS could serve as a novel multimodal prognostic biomarker for improved patient risk stratification.

## 5 Conclusions

In conclusion, through pan-cancer assessment, we demonstrated the prognostic and predictive power of MNAI in patients with different types of cancer and its association with genetic instability. Moreover, we believe that the clinical implication of MNAI and its multimodal integration with CMS will contribute to the precision prognosis and personalized treatment of cancer patients. In addition, the clinical utility of MNAI can be easily developed and implemented with an assay that simultaneously measure all 54 genes *via* different techniques, such as NanoString. The clinical utility of its multimodal integration can be achieved based on the MNAI assay as well as the adoption of the machine learning pipeline in clinical practice. In our future study, we will focus on validating MNAI as a predictive biomarker using the MNAI assay we developed for immunotherapy or PARP inhibitors and others through clinical collaborations to maximize its clinical impact.

## Data availability statement

The original contributions presented in the study are included in the article/**Supplementary Material**. Further inquiries can be directed to the corresponding author.

## Author contributions

JP-L, J-HM, and HC planned the project. HC, XM, JP-L, and J-HM wrote the manuscript. J-HM, HC, XM, YC, and SL performed the bioinformatics analyses and conducted statistical tests. All authors contributed to the article and approved the submitted version.

## References

[1] Hanahan D Weinberg RA. Hallmarks of cancer: the next generation. Cell (2011) 144(5):646–74. doi: 10.1016/j.cell.2011.02.013 21376230

[2] Takeshima H Ushijima T. Accumulation of genetic and epigenetic alterations in normal cells and cancer risk. NPJ Precis Oncol (2019) 3(1):7. doi: 10.1038/s41698-019-0079-0 30854468 PMC6403339

[3] Ha SY Choi M Lee T Park CK. The prognostic role of mitotic index in hepatocellular carcinoma patients after curative hepatectomy. Cancer Res Treat (2016) 48(1):180–9. doi: 10.4143/crt.2014.321 PMC4720078 25797572

[4] Philips P Kooby DA Maithel S Merchant NB Weber SM Winslow ER. Grading using ki-67 index and mitotic rate increases the prognostic accuracy of pancreatic neuroendocrine tumors. Pancreas (2018) 47(3):326–31. doi: 10.1097/MPA.0000000000000990 29351120

[5] Russell WO Cohen J Enzinger F Hajdu SI Heise H Martin RG. A clinical and pathological staging system for soft tissue sarcomas. Cancer (1977) 40(4):1562–70. doi: 10.1002/1097-0142(197710)40:4<1562::AID-CNCR2820400428>3.0.CO;2-6 907970

[6] Medri L Volpi A Nanni O Vecci AM Mangia A Schittulli F. Prognostic relevance of mitotic activity in patients with node-negative breast cancer. Mod Pathol (2003) 16(11):1067–75. doi: 10.1097/01.MP.0000093625.20366.9D 14614045

[7] Romansik EM Reilly CM Kass PH Moore PF London CA. Mitotic index is predictive for survival for canine cutaneous mast cell tumors. Veterinary Pathol (2007) 44(3):335–41. doi: 10.1354/vp.44-3-335 17491075

[8] Kim Y-J Ketter R Steudel W-I Feiden W. Prognostic significance of the mitotic index using the mitosis marker anti–phosphohistone H3 in meningiomas. Am J Clin Pathol (2007) 128(1):118–25. doi: 10.1309/HXUNAG34B3CEFDU8 17580279

[9] Hu Z Mao JH Curtis C Huang G Gu S Heiser L. Genome co-amplification upregulates a mitotic gene network activity that predicts outcome and response to mitotic protein inhibitors in breast cancer. Breast Cancer Res (2016) 18(1):70. doi: 10.1186/s13058-016-0728-y 27368372 PMC4930593

[10] Gaillard H García-Muse T Aguilera A. Replication stress and cancer. Nat Rev Cancer (2015) 15(5):276–89. doi: 10.1038/nrc3916 25907220

[11] Vader G Lens SM. The aurora kinase family in cell division and cancer. Biochim Biophys Acta (2008) 1786(1):60–72. doi: 10.1016/j.bbcan.2008.07.003 18662747

[12] Curtis C. Genomic profiling of breast cancers. Curr Opin Obstet Gynecol (2015) 27(1):34–9. doi: 10.1097/GCO.0000000000000145 PMC4497788 25502431

[13] Carter SL Eklund AC Kohane IS Harris LN Szallasi Z. A signature of chromosomal instability inferred from gene expression profiles predicts clinical outcome in multiple human cancers. Nat Genet (2006) 38(9):1043–8. doi: 10.1038/ng1861 16921376

[14] Strebhardt K Becker S Matthess Y. Thoughts on the current assessment of polo-like kinase inhibitor drug discovery. Expert Opin Drug Discovery (2015) 10(1):1–8. doi: 10.1517/17460441.2015.962510 25263688

[15] Kim IJ Quigley D To MD Pham P Lin K Jo B. Rewiring of human lung cell lineage and mitotic networks in lung adenocarcinomas. Nat Commun (2013) 4:1701. doi: 10.1038/ncomms2660 23591868 PMC4450149

[16] Quigley DA To MD Pérez-Losada J Pelorosso FG Mao JH Nagase H. Genetic architecture of mouse skin inflammation and tumour susceptibility. Nature (2009) 458(7237):505–8. doi: 10.1038/nature07683 PMC4460995 19136944

[17] Newman AM Liu CL Green MR Gentles AJ Feng W Xu Y. Robust enumeration of cell subsets from tissue expression profiles. Nat Methods (2015) 12(5):453–7. doi: 10.1038/nmeth.3337 PMC4739640 25822800

[18] Liu X-P Jin X Seyed Ahmadian S Yang X Tian S-F Cai Y-X. Clinical significance and molecular annotation of cellular morphometric subtypes in lower-grade gliomas discovered by machine learning. Neuro-Oncology (2022) 25(1):68–81. doi: 10.1093/neuonc/noac154 PMC9825346 35716369

[19] Chang H Yang X Moore J Liu X-P Jen K-Y Snijders AM. From mouse to human: cellular morphometric subtype learned from mouse mammary tumors provides prognostic value in human breast cancer. Front Oncol (2022) 11. doi: 10.3389/fonc.2021.819565 PMC8886672 35242697

[20] Mao X-Y Perez-Losada J Abad M Rodríguez-González M Rodríguez CA Mao J-H. iCEMIGE: integration of CEll-morphometrics, MIcrobiome, and GEne biomarker signatures for risk stratification in breast cancers. World J Clin Oncol (2022) 13(7):616–29. doi: 10.5306/wjco.v13.i7.616 PMC9346422 36157157

[21] Wenzel ES Singh ATK. Cell-cycle checkpoints and aneuploidy on the path to cancer. In Vivo (2018) 32(1):1–5. doi: 10.21873/invivo.11197 29275292 PMC5892633

[22] Levine MS Holland AJ. The impact of mitotic errors on cell proliferation and tumorigenesis. Genes Dev (2018) 32(9-10):620–38. doi: 10.1101/gad.314351.118 PMC6004076 29802124

[23] Urzúa U Ampuero S Roby KF Owens GA Munroe DJ. Dysregulation of mitotic machinery genes precedes genome instability during spontaneous pre-malignant transformation of mouse ovarian surface epithelial cells. BMC Genomics (2016) 17(8):728. doi: 10.1186/s12864-016-3068-5 27801298 PMC5088517

[24] Sun Y Liu Y Ma X Hu H. The influence of cell cycle regulation on chemotherapy. Int J Mol Sci (2021) 22(13). doi: 10.3390/ijms22136923 PMC8267727 34203270

[25] Strickler JH Hanks BA Khasraw M. Tumor mutational burden as a predictor of immunotherapy response: is more always better? Clin Cancer Res (2021) 27(5):1236–41. doi: 10.1158/1078-0432.CCR-20-3054 PMC9912042 33199494

[26] Cristescu R Aurora-Garg D Albright A Xu L Liu XQ Loboda A. Tumor mutational burden predicts the efficacy of pembrolizumab monotherapy: a pan-tumor retrospective analysis of participants with advanced solid tumors. J Immunother Cancer (2022) 10(1). doi: 10.1136/jitc-2021-003091 PMC8804694 35101941

[27] Janssen A Kops GJ Medema RH. Elevating the frequency of chromosome mis-segregation as a strategy to kill tumor cells. Proc Natl Acad Sci U.S.A. (2009) 106(45):19108–13. doi: 10.1073/pnas.0904343106 PMC2776415 19855003

[28] Ippolito MR Martis V Martin S Tijhuis AE Hong C Wardenaar R. Gene copy-number changes and chromosomal instability induced by aneuploidy confer resistance to chemotherapy. Dev Cell (2021) 56(17):2440–2454.e6. doi: 10.1016/j.devcel.2021.07.006 34352223

[29] Sharma P Pachynski RK Narayan V Fléchon A Gravis G Galsky MD. Nivolumab plus ipilimumab for metastatic castration-resistant prostate cancer: preliminary analysis of patients in the CheckMate 650 trial. Cancer Cell (2020) 38(4):489–499.e3. doi: 10.1016/j.ccell.2020.08.007 32916128

[30] Gibney GT Weiner LM Atkins MB. Predictive biomarkers for checkpoint inhibitor-based immunotherapy. Lancet Oncol (2016) 17(12):e542–51. doi: 10.1016/S1470-2045(16)30406-5 PMC5702534 27924752

[31] Ullah M Aoudjeghout W Pimpie C Pocard M Mirshahi M. Mitosis in cancer cell increases immune resistance via high expression of HLA-G and PD-L1. Cancers (Basel) (2020) 12(9). doi: 10.3390/cancers12092661 PMC7564851 32961872

[32] Kumar R Chaudhary K Gupta S Singh H Kumar S Gautam A. CancerDR: cancer drug resistance database. Sci Rep (2013) 3:1445. doi: 10.1038/srep01445 23486013 PMC3595698

[33] Chang H Zhou Y Borowsky A Barner K Spellman P Parvin B. Stacked predictive sparse decomposition for classification of histology sections. Int J Comput Vision (2014) 13(1):3–18. doi: 10.1007/s11263-014-0790-9 PMC5051579 27721567

