# Supplementary material for: Pan-cancer evaluation of clinical value of mitotic network activity index (MNAI) and its predictive value for immunotherapy
Source: Front Oncol. 2023 Jun 27;13:1178568. doi: 10.3389/fonc.2023.1178568 (PMC10349373; doi:10.3389/fonc.2023.1178568)
Supplement: Supplementary file 1 [file DataSheet_1.pdf]

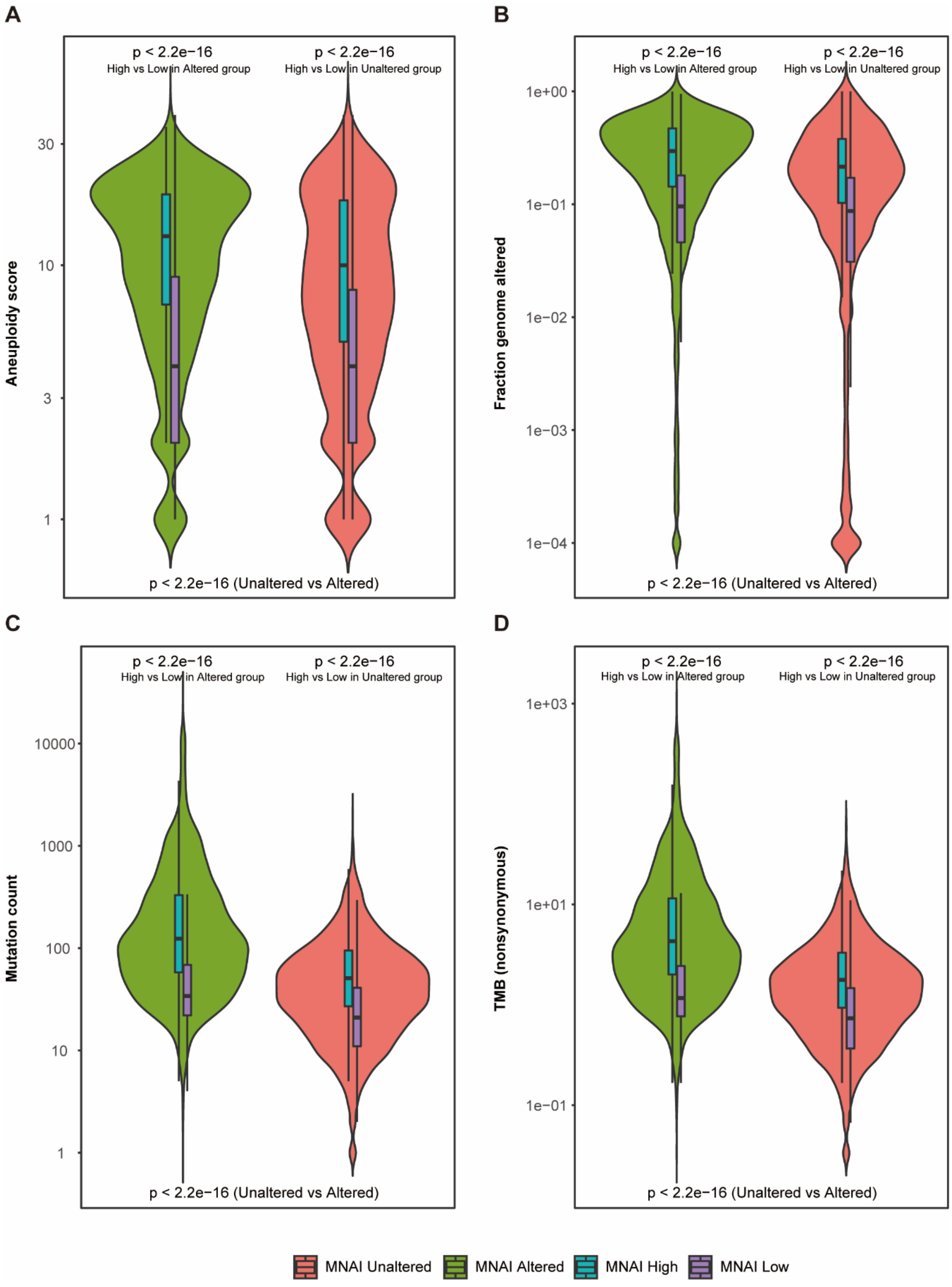


**Supplementary Figure 1**. Pan-cancer association of MNAI with genomic instability. (A) Aneuploidy score; (B) Fraction genome altered; (C) Mutation count; (D) Tumor mutation burden (TMB, nonsynonymous). The p values were obtained from Wilcoxon test.

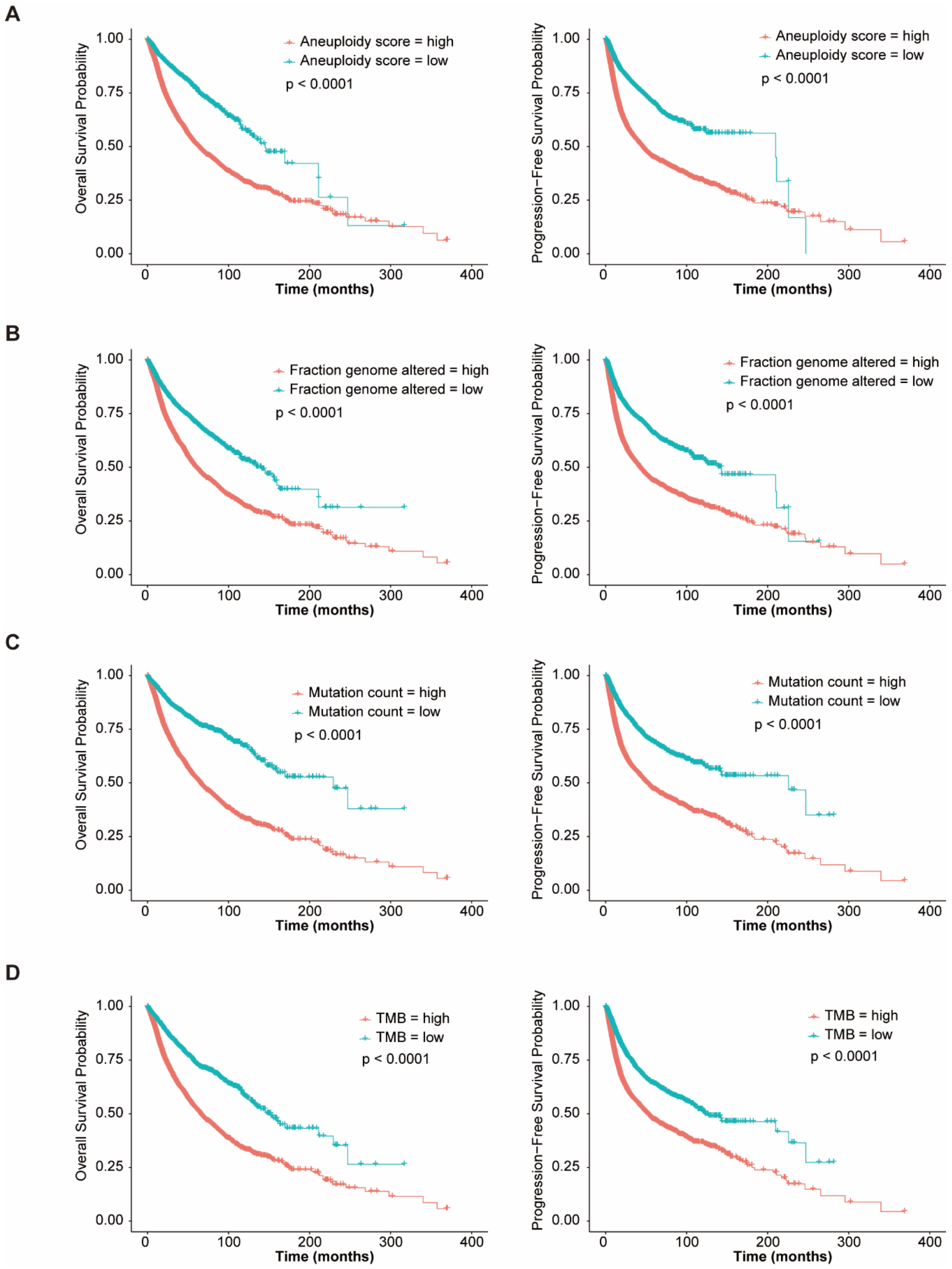


**Supplementary Figure 2**. Pan-cancer prognostic value of genomic instability on OS and PFS in TCGA. (A) Aneuploidy score; (B) Fraction genome altered; (C) Mutation count; (D) Tumor mutation burden (TMB, nonsynonymous). The p values were obtained from log-rank test.

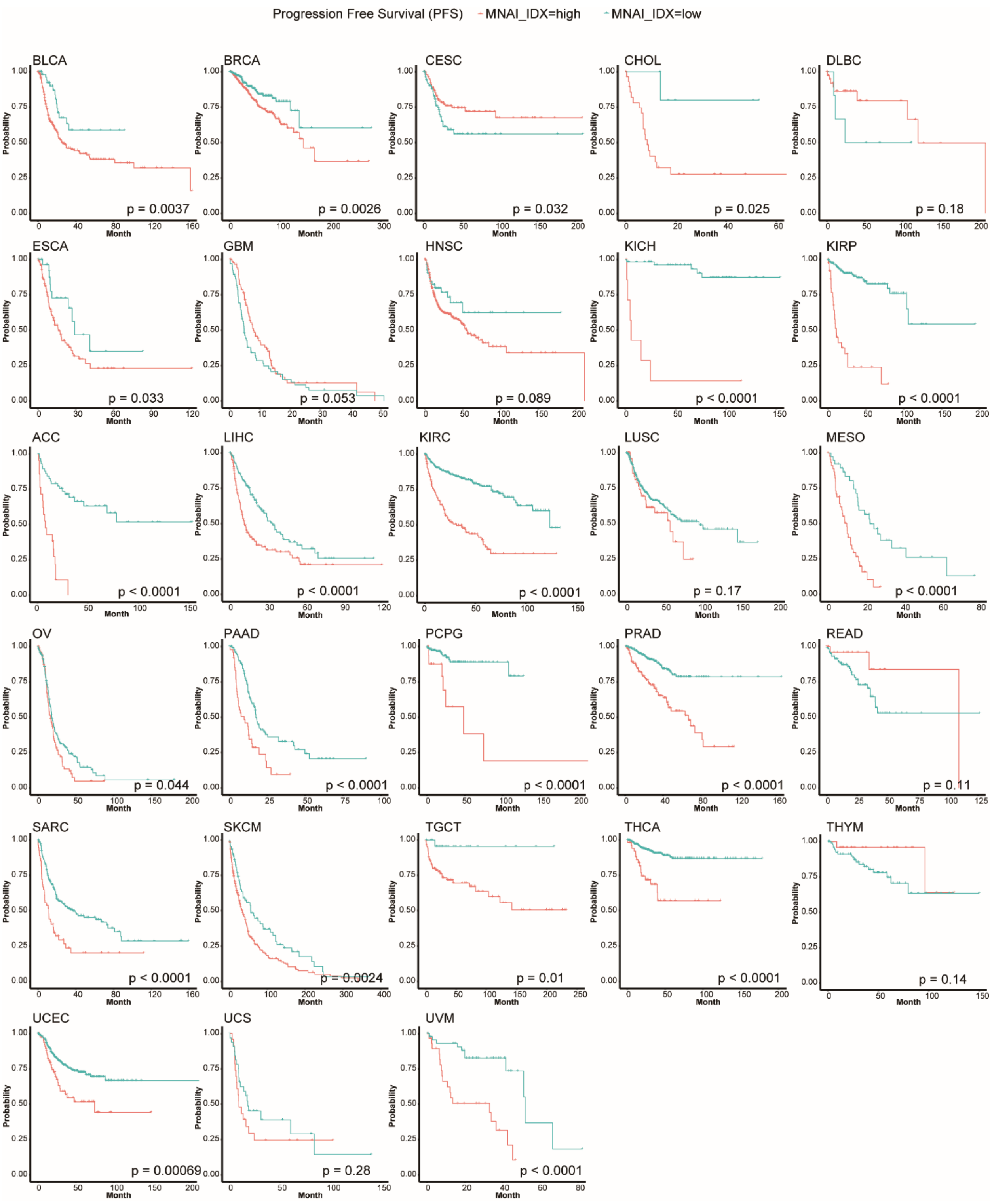


**Supplementary Figure 3**. Pan-cancer prognostic value of MNAI on PFS in TCGA, where the cutoff points were obtained towards PFS using surv_cutpoint function in R and the p values were obtained using log-rank test.

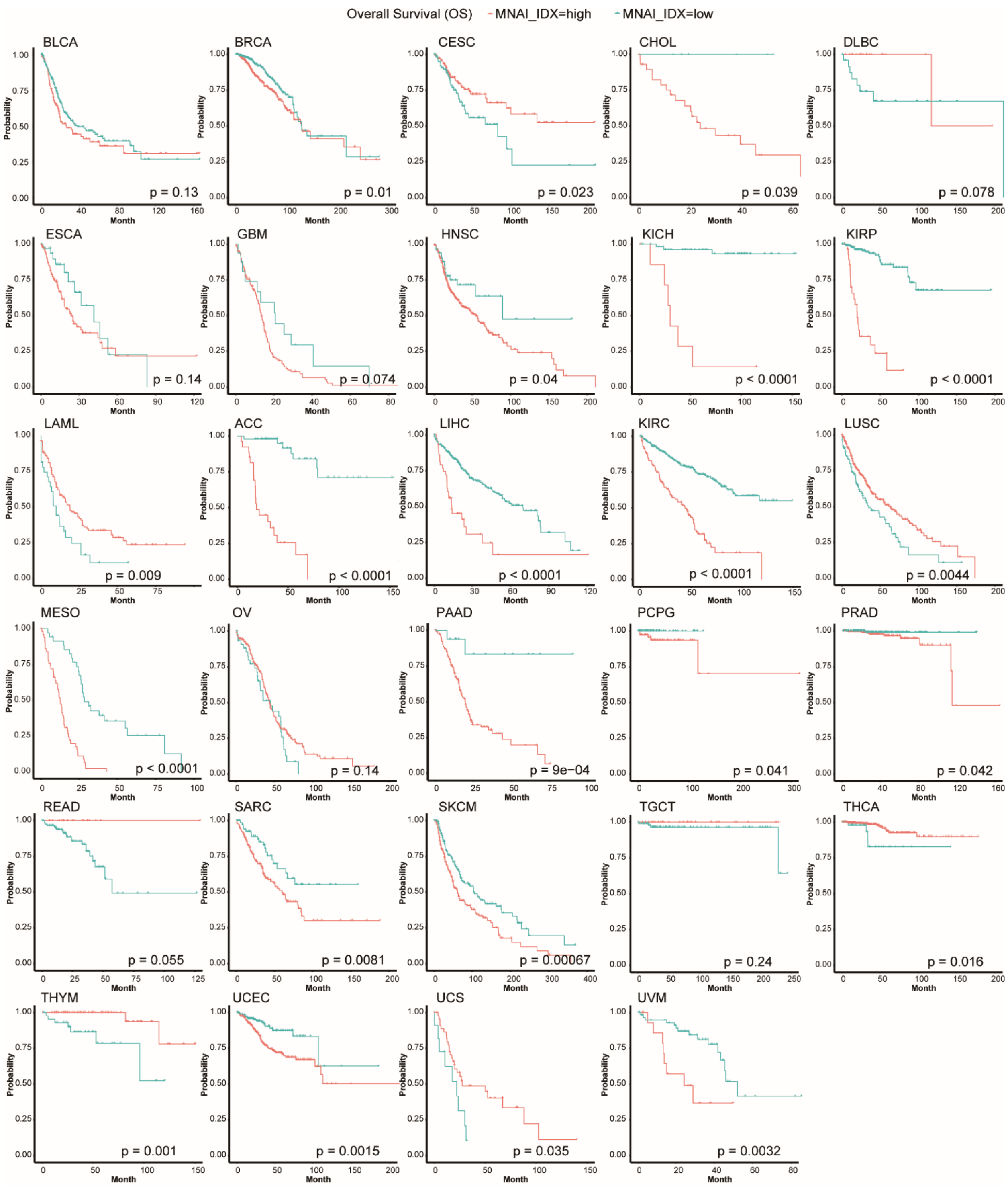


**Supplementary Figure 4**. Pan-cancer prognostic value of MNAI on OS in TCGA, where the cutoff points were obtained towards OS using surv_cutpoint function in R and the p values were obtained using log-rank test.

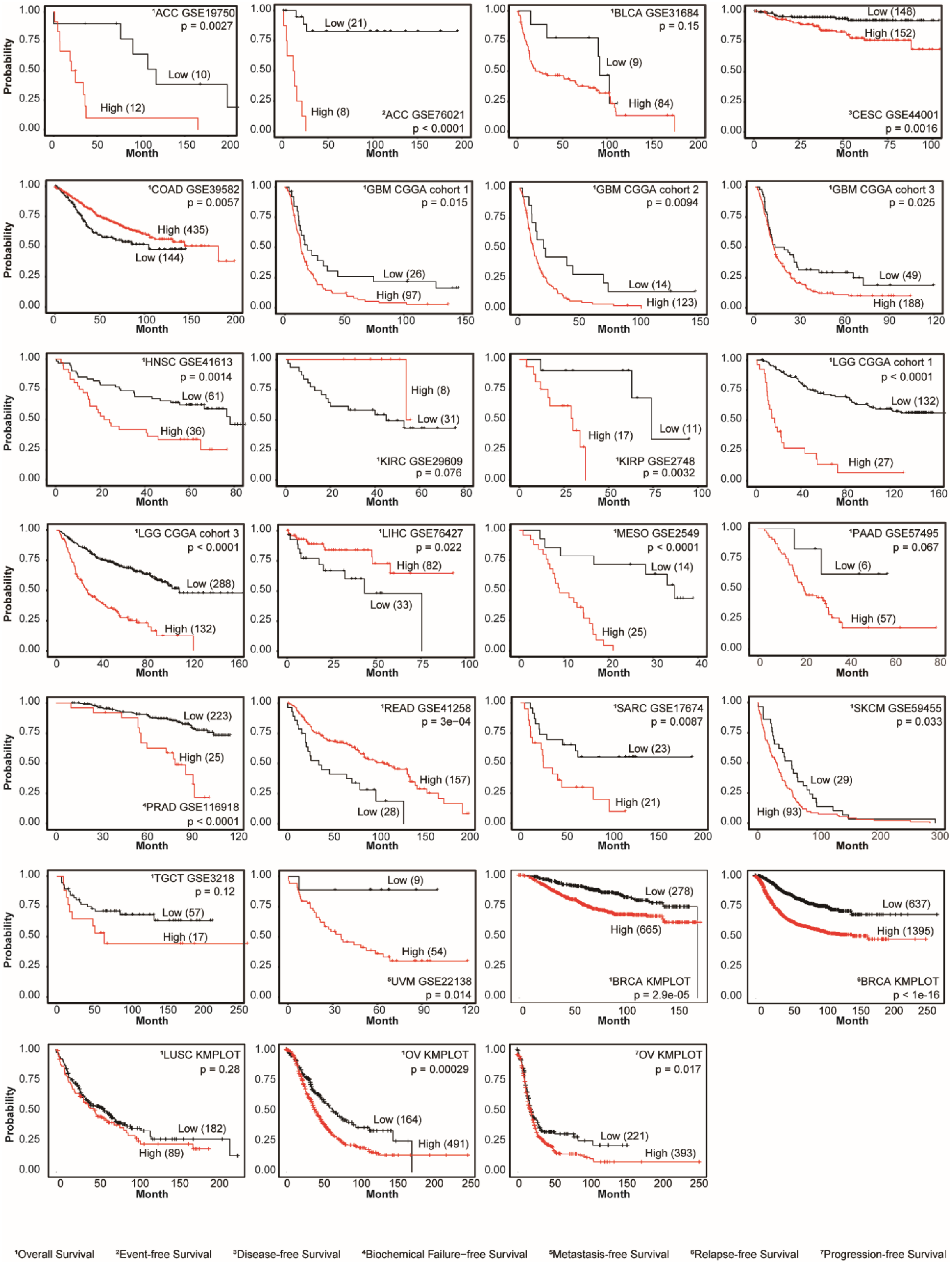


**Supplementary Figure 5**. Pan-cancer prognostic value of MNAI on different prognostic endpoints in validation cohorts, where the cutoff points were obtained towards the target prognostic endpoints using surv_cutpoint function in R and the p values were obtained using log-rank test.

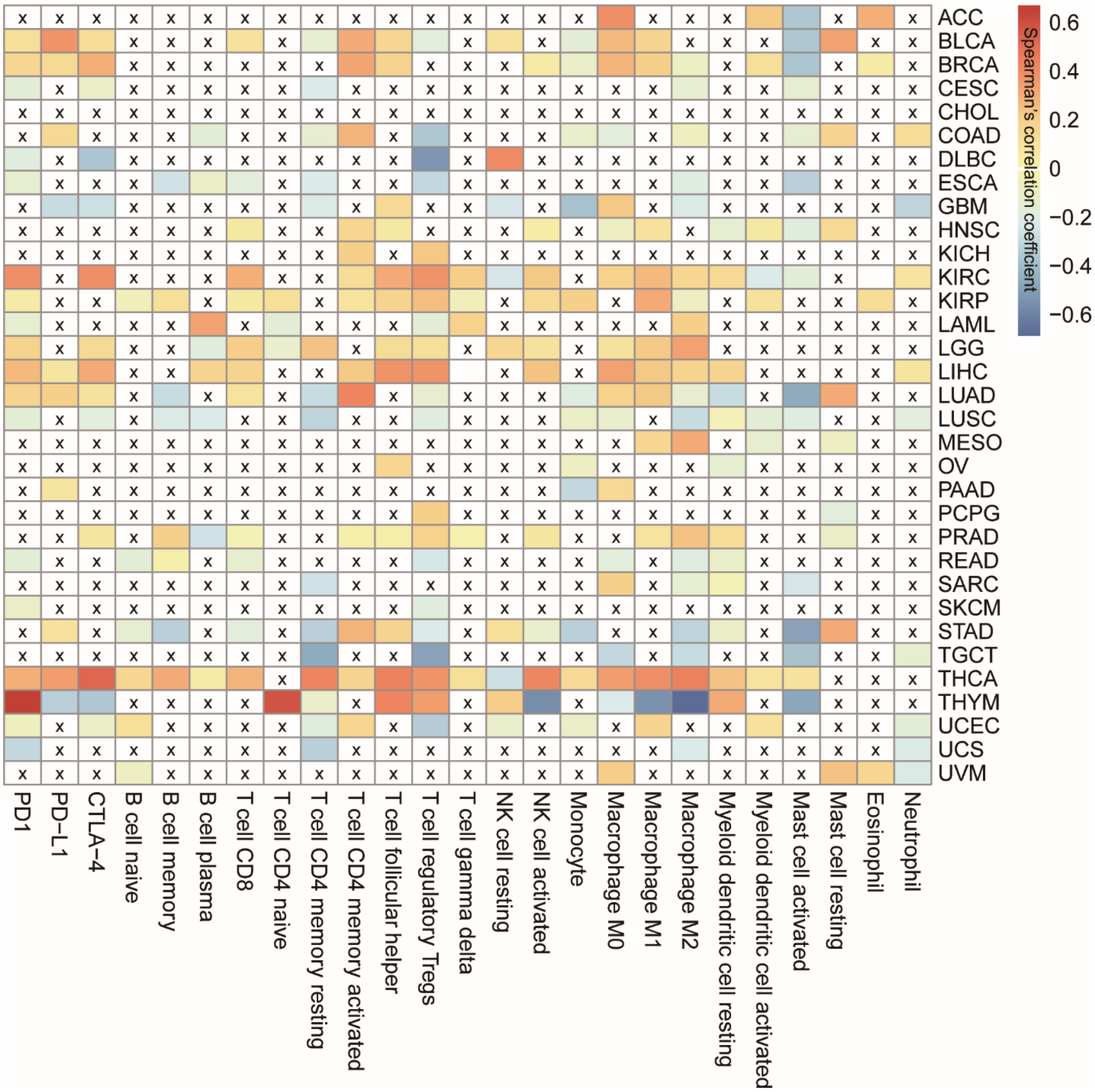


**Supplementary Figure 6**. Association of MNAI with PD1, PD-L1, CTLA-4 and some immune cell infiltrations across tumor types in TCGA. The association was assessed using spearman's correlation, where "x" marks the non-significant (p>=0.05) associations. The abundance of different cell types were estimated using CIBERSORT in absolute mode. The color bar encodes the value of spearman's correlation coefficients.

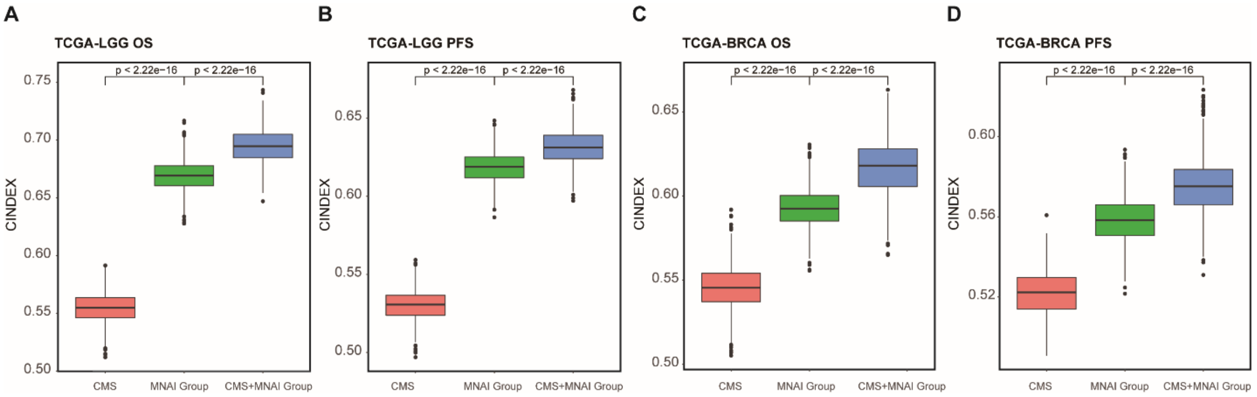


**Supplementary Figure 7**. Multimodal integration of MNAI and CMS exceeds individual models towards precision prognosis in TCGA-LGG (A-B) and TCGA-BRCA (C-D) cohorts. Specifically, concordance index (C-index) were used to evaluate the performance of the CMS, MNAI and integrated models, with 1000 bootstrapping iterations and an 80% sampling rate per iteration. Mann-Whitney non-parametric test was used for the comparison across models

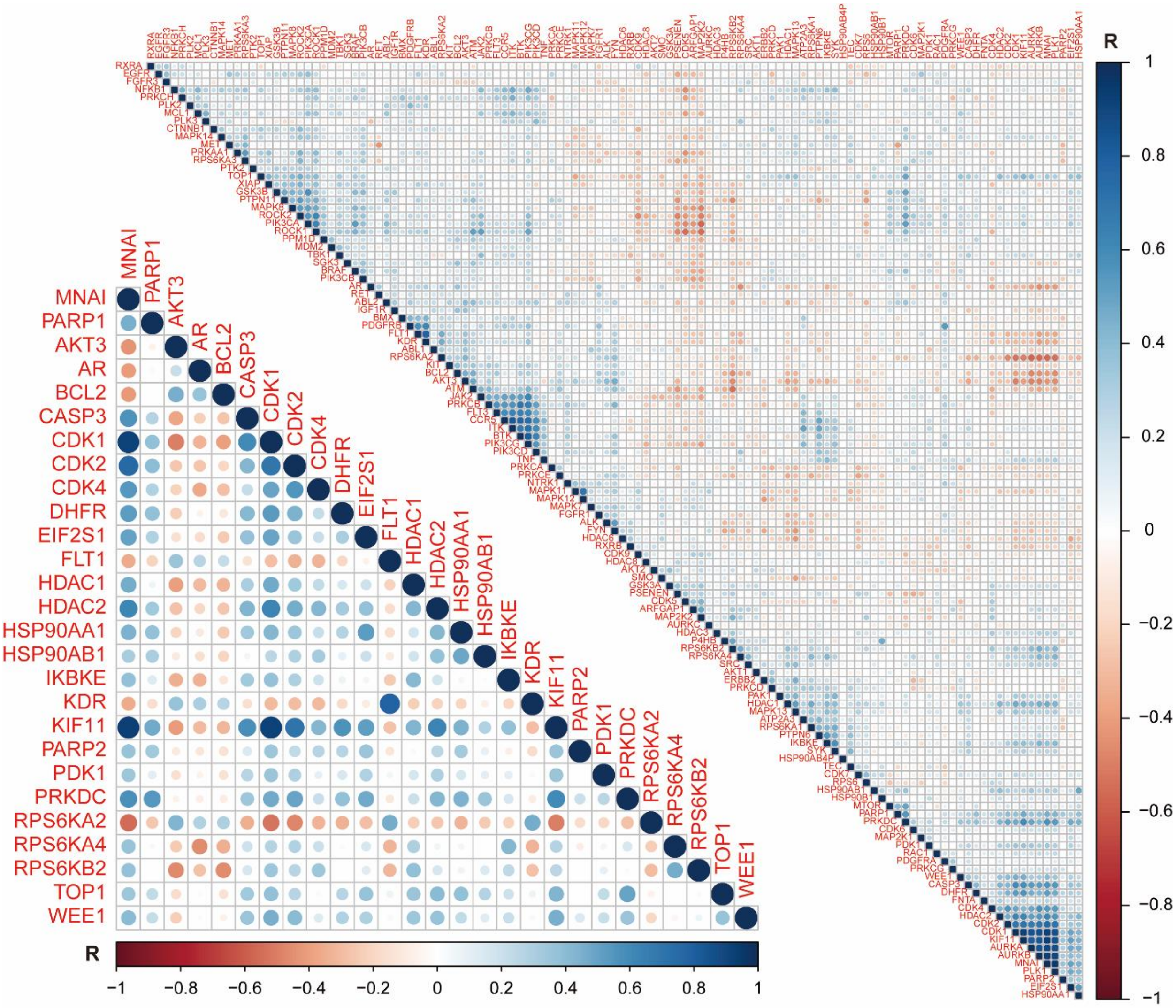


**Supplementary Figure 8**. TCGA Pan-cancer association of MNAI with different molecular targets obtained from CancerDR database. Upper pane, correlation plots with all molecular targets available in both CancerDR and TCGA; Lower pane, correlation plots with significant molecular targets (Spearman correlation, |R|>0.3 and p value < 0.05) available in both CancerDR and TCGA.

**Supplementary Table 1**. Information on TCGA datasets used in the study.

| Study Abbreviation | Study Name | Number of OS events | Number of PFS events | Total number of patients |
|---|---|---|---|---|
| **ACC** | Adrenocortical carcinoma | 27 | 40 | 78 |
| **BLCA** | Bladder Urothelial Carcinoma | 179 | 174 | 407 |
| **BRCA** | Breast invasive carcinoma | 151 | 145 | 1082 |
| **CESC** | Cervical squamous cell carcinoma and endocervical adenocarcinoma | 68 | 68 | 294 |
| **CHOL** | Cholangiocarcinoma | 18 | 20 | 36 |
| **COAD** | Colon adenocarcinoma | 98 | 116 | 436 |
| **DLBC** | Lymphoid Neoplasm Diffuse Large B-cell Lymphoma | 9 | 12 | 48 |
| **ESCA** | Esophageal carcinoma | 76 | 85 | 181 |
| **GBM** | Glioblastoma multiforme | 122 | 121 | 155 |
| **HNSC** | Head and Neck squamous cell carcinoma | 216 | 194 | 515 |
| **KICH** | Kidney Chromophobe | 9 | 11 | 65 |
| **KIRC** | Kidney renal clear cell carcinoma | 168 | 158 | 510 |
| **KIRP** | Kidney renal papillary cell carcinoma | 41 | 55 | 283 |
| **LAML** | Acute Myeloid Leukemia | 114 | NA | 173 |
| **LGG** | Brain Lower Grade Glioma | 125 | 192 | 514 |
| **LIHC** | Liver hepatocellular carcinoma | 129 | 177 | 366 |
| **LUAD** | Lung adenocarcinoma | 185 | 206 | 510 |
| **LUSC** | Lung squamous cell carcinoma | 207 | 140 | 484 |
| **MESO** | Mesothelioma | 74 | 61 | 87 |
| **OV** | Ovarian serous cystadenocarcinoma | 180 | 207 | 300 |
| **PAAD** | Pancreatic adenocarcinoma | 92 | 103 | 177 |
| **PCPG** | Pheochromocytoma and Paraganglioma | 5 | 20 | 178 |
| **PRAD** | Prostate adenocarcinoma | 10 | 93 | 493 |
| **READ** | Rectum adenocarcinoma | 21 | 32 | 154 |
| **SARC** | Sarcoma | 96 | 137 | 253 |
| **SKCM** | Skin Cutaneous Melanoma | 212 | 303 | 441 |
| **STAD** | Stomach adenocarcinoma | 159 | 134 | 412 |
| **TGCT** | Testicular Germ Cell Tumors | 4 | 34 | 149 |
| **THCA** | Thyroid carcinoma | 16 | 51 | 497 |
| **THYM** | Thymoma | 9 | 21 | 119 |
| **UCEC** | Uterine Corpus Endometrial Carcinoma | 87 | 119 | 527 |
| **UCS** | Uterine Carcinosarcoma | 35 | 37 | 57 |
| **UVM** | Uveal Melanoma | 23 | 30 | 80 |

**Supplementary Table 2.** Datasets used in validation study

| Tumor Type Abbreviation | Tumor Type | Dataset | Clinical Endpoint | Total number of patients |
|---|---|---|---|---|
| ACC | Adrenocortical carcinoma | GSE19750 | overall survival | 22 |
| | | GSE76021 | event-free survival | 29 |
| BLCA | Bladder Urothelial Carcinoma | GSE31684 | overall survival | 93 |
| BRCA | Breast invasive carcinoma | [#]KMPLOT | overall survival | 943 |
| | | | relapse-free survival | 2032 |
| CESC | Cervical squamous cell carcinoma and endocervical adenocarcinoma | GSE44001 | disease-free survival | 300 |
| COAD | Colon adenocarcinoma | GSE39582 | overall survival | 582 |
| GBM | Glioblastoma multiforme | CGGA Cohort 1 | overall survival | 123 |
| | | CGGA Cohort 2 | overall survival | 137 |
| | | CGGA Cohort 3 | overall survival | 237 |
| HNSC | Head and Neck squamous cell carcinoma | GSE41613 | overall survival | 97 |
| KIRC | Kidney renal clear cell carcinoma | GSE29609 | overall survival | 39 |
| KIRP | Kidney renal papillary cell carcinoma | GSE2748 | overall survival | 28 |
| LGG | Brain Lower Grade Glioma | CGGA Cohort 1 | overall survival | 159 |
| | | CGGA Cohort 2 | overall survival | 172 |
| | | CGGA Cohort 3 | overall survival | 420 |
| LIHC | Liver hepatocellular carcinoma | GSE76427 | overall survival | 115 |
| LUAD | Lung adenocarcinoma | KMPLOT | overall survival | 672 |
| LUSC | Lung squamous cell carcinoma | KMPLOT | overall survival | 271 |
| MESO | Mesothelioma | GSE2549 | overall survival | 39 |
| OV | Ovarian cancer | KMPLOT | overall survival | 655 |
| | | | progression-free survival | 614 |
| PAAD | Pancreatic adenocarcinoma | GSE57495 | overall survival | 63 |
| PRAD | Prostate adenocarcinoma | GSE116918 | Biochemical failure-free survival | 248 |
| READ | Rectum adenocarcinoma | GSE41258 | overall survival | 185 |
| SARC | Sarcoma | GSE17674 | overall survival | 44 |

| | | | | |
|---|---|---|---|---|
| SKCM | Skin Cutaneous Melanoma | GSE59455 | overall survival | 122 |
| STAD | Gastric cancer | KMPLOT | overall survival | 631 |
| TGCT | Testicular Germ Cell Tumors | GSE3218 | overall survival | 74 |
| UVM | Uveal Melanoma | GSE22138 | Metastasis free survival | 63 |

#meta-analysis was carried out using Kaplan-Meier Plotter (http://kmplot.com/analysis/)
